# Use of a Lymphatic Drug Delivery System and Sonoporation to Target Malignant Metastatic Breast Cancer Cells Proliferating in the Marginal Sinuses

**DOI:** 10.1038/s41598-019-49386-5

**Published:** 2019-09-13

**Authors:** Shigeki Kato, Yuko Shirai, Maya Sakamoto, Shiro Mori, Tetsuya Kodama

**Affiliations:** 1 Laboratory of Biomedical Engineering for Cancer, Graduate School of Biomedical Engineering, Tohoku University, 4-1 Seiryo, Aoba, Sendai, Miyagi 980-8575 Japan; 2 Biomedical Engineering Cancer Research Center, Graduate School of Biomedical Engineering, Tohoku University, 4-1 Seiryo, Aoba, Sendai, Miyagi 980-8575 Japan; 3 Department of Immunology, Kindai University Faculty of Medicine, Osaka-Sayama, Osaka, 589-8511 Japan; 4 Department of Oral Diagnosis, Tohoku University Hospital, 1-1 Seiryo, Aoba, Sendai, Miyagi 980-8575 Japan; 5 Department of Oral Medicine and Surgery, Tohoku University Hospital, 1-1 Seiryo, Aoba, Sendai, Miyagi 980-8575 Japan

**Keywords:** Targeted therapies, Biomedical engineering

## Abstract

Lymph node (LN) metastasis through the lymphatic network is a major route for cancer dissemination. Tumor cells reach the marginal sinuses of LNs via afferent lymphatic vessels (LVs) and form metastatic lesions that lead to distant metastasis. Thus, targeting of metastatic cells in the marginal sinuses could improve cancer treatment outcomes. Here, we investigated whether lymphatic administration of a drug combined with sonoporation could be used to treat a LN containing proliferating murine FM3A breast cancer cells, which are highly invasive, in its marginal sinus. First, we used contrast-enhanced high-frequency ultrasound and histopathology to analyze the structure of LVs in MXH10/Mo-*lpr*/*lpr* mice, which exhibit systemic lymphadenopathy. We found that contrast agent injected into the subiliac LN flowed into the marginal sinus of the proper axillary LN (PALN) and reached the cortex. Next, we examined the anti-tumor effects of our proposed technique. We found that a strong anti-tumor effect was achieved by lymphatic administration of doxorubicin and sonoporation. Furthermore, our proposed method prevented tumor cells in the marginal sinus from invading the parenchyma of the PALN and resulted in tumor necrosis. We conclude that lymphatic administration of a drug combined with sonoporation could exert a curative effect in LNs containing metastatic cells in their marginal sinuses.

## Introduction

Breast cancer is a leading cause of death in women in Japan and other countries^1^. In the advanced stages of breast cancer, malignant cells often invade the lymphatic vessels (LVs) and migrate to downstream lymph nodes (LNs), and this usually indicates a poor prognosis for the patient^2,3^. During lymphatic metastasis, metastatic lesions first form in the marginal sinuses of the LNs, and the tumor cells then spread to distant organs through the lymphatic network and/or blood circulation^4^. Thus, metastatic cancer cells in the marginal sinus are a potential therapeutic target to improve prognosis. However, a radical treatment for metastatic tumor cells in the marginal sinus has yet to be established unequivocally.

**Figure 1:**
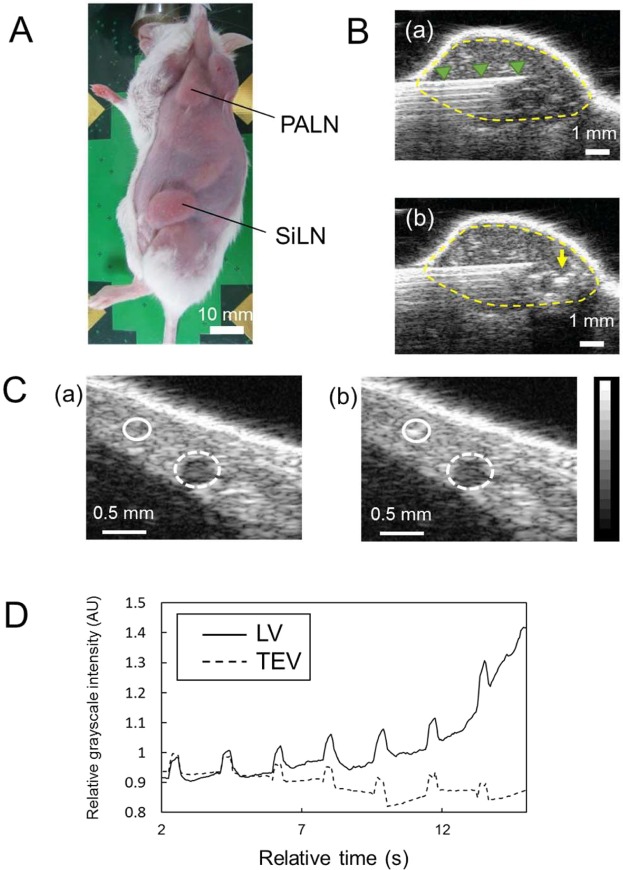
Lymphatic administration of acoustic liposomes (ALs) in MXH10/Mo/lpr mice and analysis of the kinetics of ALs in the lymphatic vessel (LV) using high-frequency ultrasound (HF-US) imaging. (**A**) Schematic view of a MXH10/Mo/lpr mouse, which has swollen lymph nodes (LNs) comparable in size to those in humans. The subiliac LN (SiLN) and proper axillary LN (PALN) are connected by the LV located under the skin. PALN: proper axillary lymph node; SiLN: subiliac lymph node. (**B**) Injection of ALs into the SiLN. (a) A butterfly needle was inserted into the SiLN under the guidance of the HF-US system. The arrowheads indicate the butterfly needle inserted into the SiLN. (b) After the injection of ALs into the SiLN, the echogenicity of the ALs was detected in the SiLN. The arrow indicates the echogenicity of the ALs administered into the SiLN. The dotted line represents the border of the SiLN. (**C**) Representative B-mode images of the TEV (dotted circle) and LV (solid circle) located between the SiLN and PALN. (a) Echogenicity was not detected in either vessel before the administration of ALs. (b) When ALs were flowing through the field of view, echogenicity was detected in the LV but not TEV. (**D**) Time-dependent changes in relative grayscale intensity in the LV and TEV. Grayscale intensity in the LV increased as ALs passed through the vessel. Grayscale intensity in the TEV was maintained at a low level because most of the ALs injected into the SiLN did not migrate into the TEV. The B-mode images were acquired from a 15.0 mm × 15.0 mm area at a frame rate of 15 Hz. Solid line: LV; dotted line: TEV.

Treatments such as surgical dissection, radiation, and chemotherapy have limitations associated with high invasiveness and severe adverse events, which can lead to the cessation of continuing therapy. We previously demonstrated that lymphatic administration of drugs combined with sonoporation using acoustic liposomes (ALs) and ultrasound (US) was a promising method for treating a tumor-bearing LN^5,6^. We have used MXH10/Mo-*lpr*/*lpr* (MXH10/Mo/lpr) mice to establish a preclinical mouse model of lymphatic drug administration to a tumor-bearing LN. These mice exhibit systemic lymphadenopathy, resulting in LNs the sizes of which are similar to those in humans (up to 10 mm; Fig. 1A)^7^. US imaging-guided injection of a drug into a LN enabled the drug to flow into the LVs (Fig. 1B). Thus, after injection into the subiliac LN (SiLN), the drug could reach the proper axillary LN (PALN) via the LVs^8^. US-mediated drug delivery using ALs and sonoporation generates mechanical pressures and induces cell membrane permeability, which permits foreign molecules to enter live cells without the occurrence of tissue damage or immune responses^9–15^. One advantage of US-mediated drug delivery is that repeated sonoporation facilitates enhanced drug delivery into target cells without significant tissue damage^16^.

However, in our previous research evaluating the effects of lymphatic drug delivery and sonoporation, the mouse model of LN metastasis was generated using KM-Luc/GFP cells, which have low invasive growth characteristics and form tumor regions with well-defined borders in or near the marginal sinuses. No studies published to date have investigated whether lymphatic administration of an anticancer agent with sonoporation would be effective against highly invasive tumor cells such as the FM3A murine breast cancer cell line. FM3A cells have high invasive growth characteristics, proliferate along the trabecular sinus and invade the cortex and paracortex^17^. Conventional chemotherapeutic strategies fail to deliver drugs into tumor masses located in the marginal and lymphatic sinuses because the sinuses have a poor blood supply. One promising strategy to overcome these obstacles would be lymphatic administration of drugs combined with sonoporation, and we have applied this method successfully against tumor masses in LNs formed by KM-Luc/GFP cells^5^. However, no previous study has evaluated whether this treatment strategy would be effective against highly invasive tumor cells that show a tendency to proliferate along the marginal and lymphatic sinuses of metastatic LNs.

To this end, the main aim of the present study was to demonstrate that lymphatic drug administration combined with sonoporation could be an effective therapy for invasive tumor cells in the marginal sinuses of LNs. Accordingly, a tumor-bearing LN model was developed in MXH10/Mo/lpr mice using a murine breast cancer cell line (FM3A-Luc cells) stably expressing the luciferase firefly gene. First, we visualized and analyzed the kinetics of ALs in the LVs and LNs of MXH10/Mo/lpr mice using a high-frequency US (HF-US) imaging system. Next, we investigated the anti-tumor effects of lymphatic drug administration combined with sonoporation against intranodal tumor cells, which had grown and proliferated in the marginal sinuses.

## Results

### Visualization of the thoracoepigastric vein (TEV) and LVs with contrast-enhanced HF-US and lymphatic administration of ALs

ALs were injected into the SiLN, and the dynamics of the flow of ALs into the LVs were analyzed using contrast-enhanced HF-US. Figure 1C shows representative B-mode images in the lateral position before (Fig. 1C(a)) and after (Fig. 1C(b)) the injection of ALs. When the ALs passed through the field of view, increasing echogenicity was detected in the LV (solid circle) but not in the TEV (dotted circle). Figure 1D shows the time-dependent changes in relative grayscale intensity in the LV and TEV. The grayscale intensity of the LV gradually increased, while that in the TEV showed no changes throughout the experiment.

### Quantification of LV density in the PALN by contrast-enhanced HF-US and 3-D imaging of the PALN

**Figure 2:**
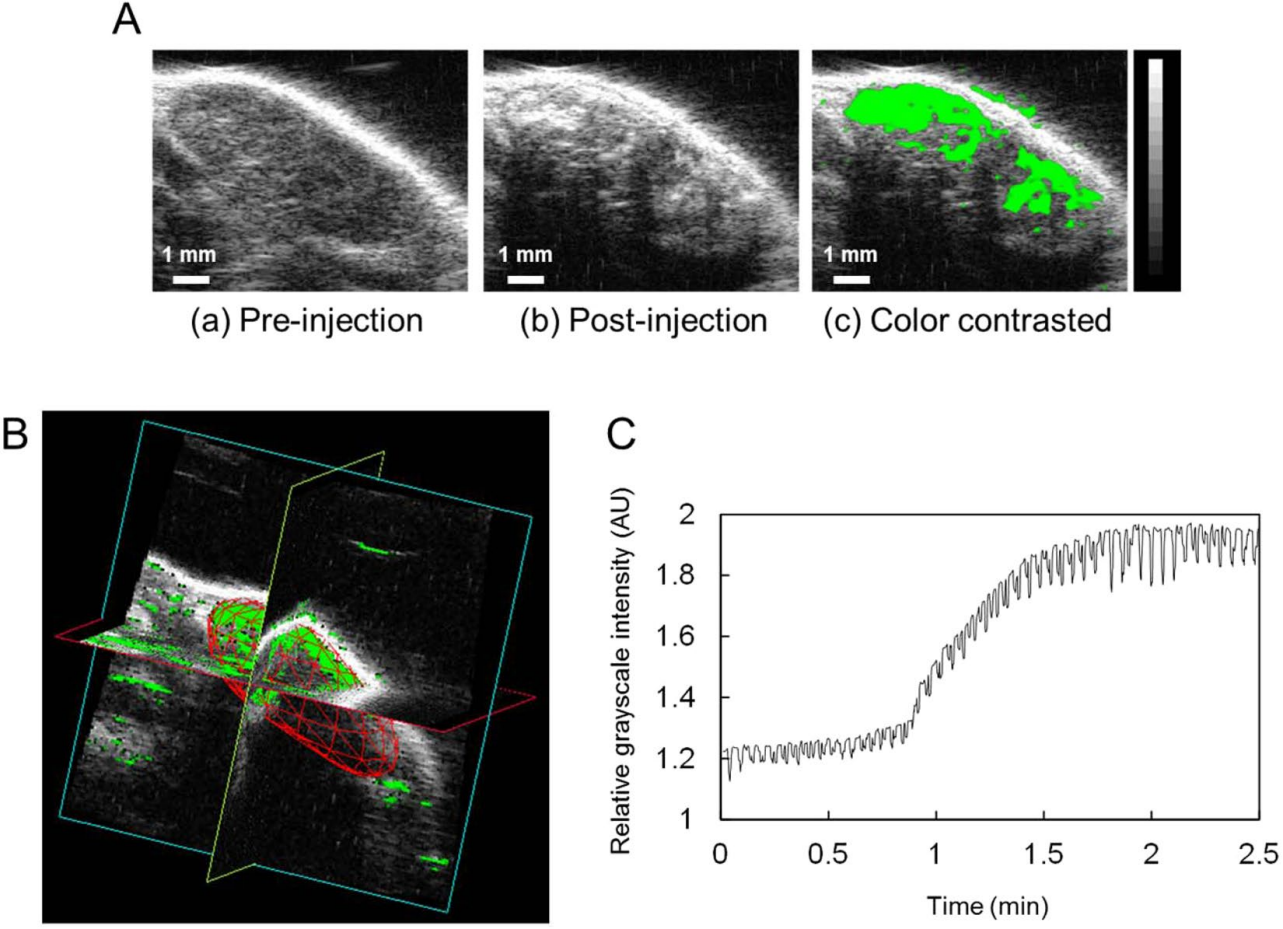
Detection of acoustic liposomes (ALs) in the proper axillary lymph node (PALN) using the high-frequency ultrasound (HF-US) system. (**A**) (a) Representative B-mode image of the PALN before the injection of ALs. (b) B-mode image of the same section as (a) after the injection of 150 μL of ALs into the subiliac lymph node (SiLN). (c) The green-colored region represents the difference between (a) and (b), indicating lymphatic vessels (LVs) in the PALN. The green-colored peripheral region represents the marginal sinus and the central region indicates the cortex of the PALN. (**B**) A representative 3-D image of the PALN after the injection of ALs. The LVs (colored green) were localized to the peripheral region, which is the marginal sinus. (**C**) Time-dependent changes in the relative grayscale intensity in the PALN. The grayscale intensity started to increase gradually after about 50 sec and became constant at an elevated level at around 2 min after the injection of ALs.

The density of LVs in the PALN was calculated using the contrast enhanced HF-US system, and 3-D images of the PALN were generated. Figure 2A shows representative B-mode images of the PALN before (Fig. 2A(a)) and after (Fig. 2A(b)) the administration of ALs. The echogenicity of the ALs was observed in the marginal sinus and could be detected around the cortical region of the PALN. The green-colored area in Fig. 2A(c) illustrates the increase in echogenicity between Fig. 2A(b) and Fig. 2A(a). To construct a 3-D image, every B-mode image was integrated into a whole PALN as depicted in Fig. 2B. The green areas, which indicate the LVs, were localized to a peripheral region that was the marginal sinus. The volume and LV density of the PALN were 173.98 ± 45.95 mm^3^ and 31.91 ± 4.2%, respectively (Table 1). Time-dependent changes in the relative grayscale intensity of the PALN are depicted in Fig. 2C. The grayscale intensity started to increase gradually after 1 min, reached a maximum at around 2 min and then remained constant.

**Table 1:** Volume and LV density of the PALN.

PALN volume (mm^3^)	% of LV area in PALN
173.98 ± 45.95	31.91 ± 4.2

### Distribution of lymphatically-administered India ink in the tumor-bearing PALN

**Figure 3:**
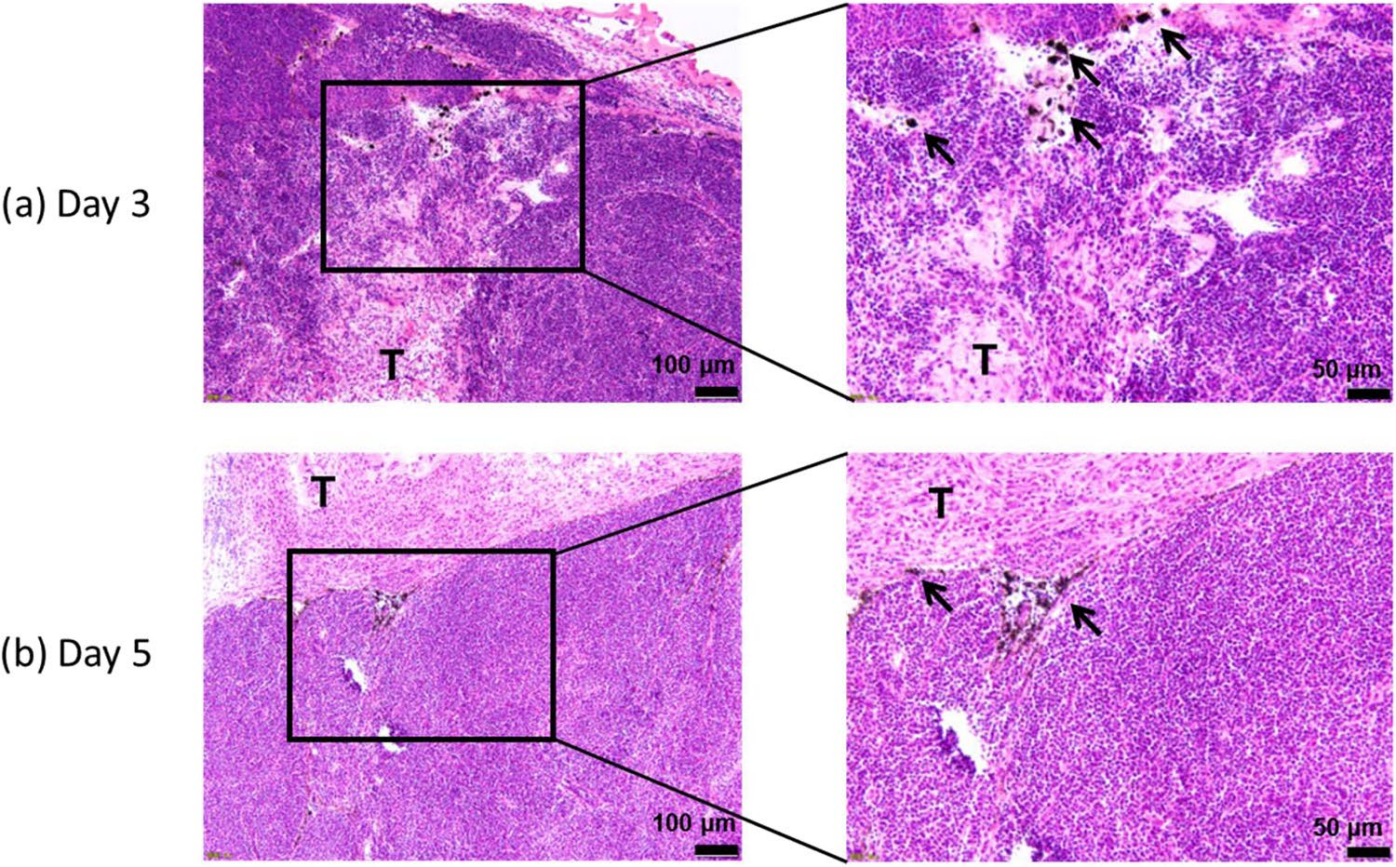
Distribution of India ink in the tumor-bearing proper axillary lymph node (PALN). Hematoxylin/eosin-stained sections of the PALN. Three days (**a**) or 5 days (**b**) after tumor cell inoculation, India ink was injected into the SiLN and reached the PALN through the upstream lymphatic vessels (LVs). Tumor cells had proliferated in the cortex and invaded the parenchyma of the PALN. India ink (arrows) was detected near the tumor lesion (T) in the PALN.

A histopathological analysis was performed to demonstrate whether a solution was able to reach the tumor-bearing PALN through LVs. Figure 3a shows representative images of the tumor-bearing PALN on day 3 (Fig. 3(a)) and day 5 (Fig. 3(b)) after tumor cell inoculation. The right-hand panels are enlarged sections of the left-hand panels. Tumor lesions were observed around the medulla, and India ink was detected in the lymphatic sinus near the medulla on day 3. At 5 days post-inoculation, cancer cells had invaded the lymphatic sinus and expanded into the parenchyma between the cortex and lymphatic sinus, where India ink could be detected.

### Evaluation of the anti-tumor effects of lymphatic drug administration combined with sonoporation in a murine breast tumor-bearing LN

**Figure 4:**
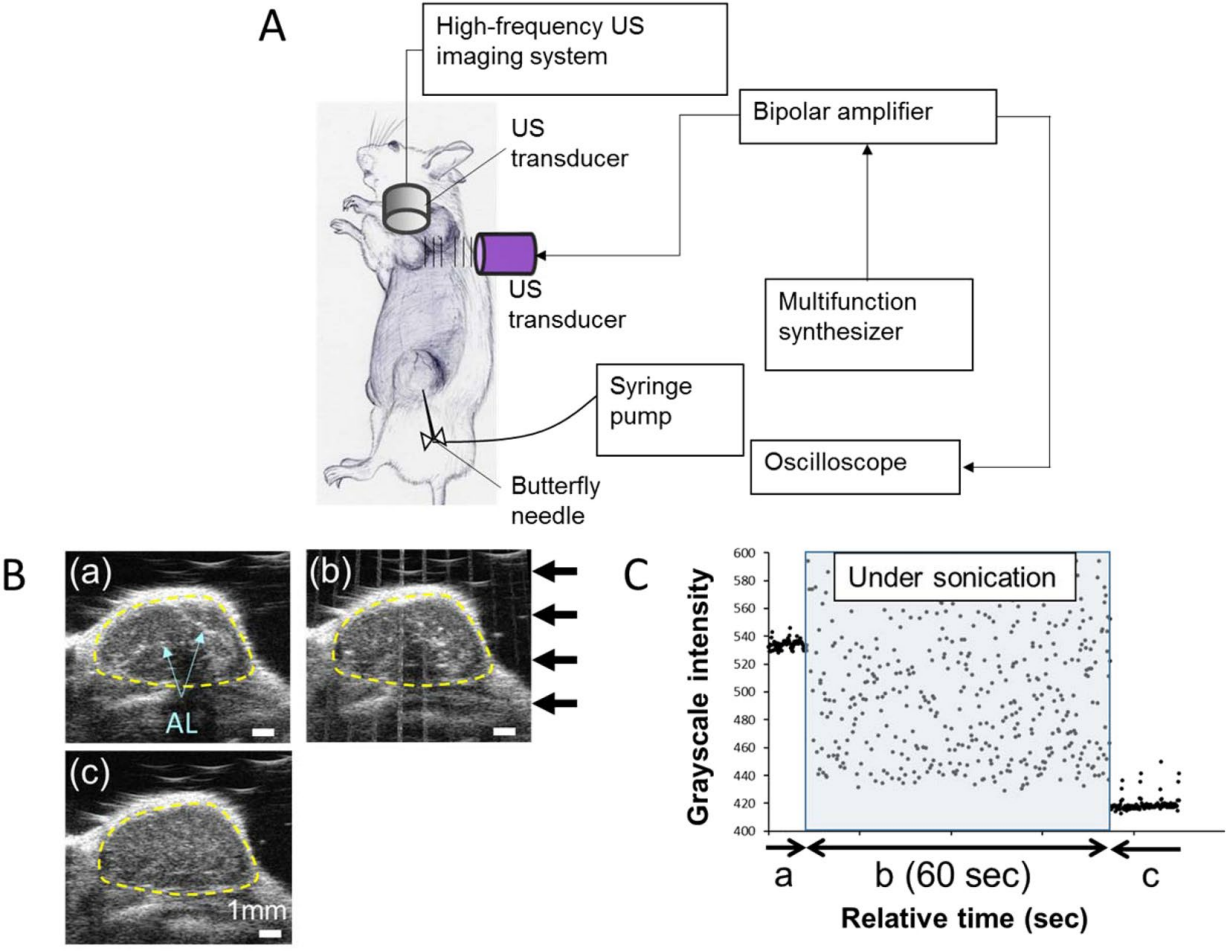
Delivery of a drug to a tumor-bearing lymph node (LN) via the lymphatic vessels (LVs) and sonication. (**A**) The position of the high-frequency ultrasound (HF-US) imaging transducer was fixed after identification of the vein known to run parallel to the LV. Solutions consisting of acoustic liposomes (ALs) and doxorubicin (DOX) were injected into the subiliac LN (SiLN) at a constant rate (using a driven syringe pump), and the US transducer for sonoporation was moved to the proper axillary LN (PALN) after confirmation that ALs were running through the LVs in the lateral region. Immediately after finishing the administration of the solutions, the PALN was exposed to US. The US signals were generated by a multifunction synthesizer and amplified by a bipolar amplifier. During all applications of US, the US output was confirmed using a contrast enhanced HF-US imaging system and oscilloscope. (**B**) Temporal changes in echogenicity in the PALN and destruction of the ALs by exposure to US. (a) After the injection of DOX with ALs into the SiLN at a rate of 50 μL/min, ALs were detected in the lymphatic channel. (b) Instantaneous image taken during irradiation showing disappearance of the echogenicity of the ALs after irradiation. The intensity of the spatial peak and temporal average of the US was 2.93 W/cm^2^ under these conditions. The arrows represent the direction of the destructive US. (c) After irradiation of the PALN, no echogenicity was detected in the PALN due to destruction of the ALs. (**C**) Grayscale intensity of the PALN before sonication (a), during sonication for 60 seconds (b) and after sonication (c).

The procedures used for lymphatic drug administration and sonoporation are illustrated in Fig. 4A. The PALN was monitored using a contrast-enhanced HF-US imaging system to confirm whether co-administered doxorubicin (DOX) and ALs reached the PALN. Before administration of the solutions, echogenicity was not detected in the PALN. About 60 sec after administration of the solutions, the echogenicity of the ALs was detected in the peripheral region of the PALN, i.e., the marginal sinus. Subsequently, the echogenicity in the PALN increased as the ALs flowed into the cortex (Fig. 4B(a)). US was applied to the PALN (Fig. 4B(b)) after the injection of ALs had been completed to induce the collapse of the ALs (Fig. 4B(c)). The ALs from the afferent LVs of the PALN flowed into the marginal sinuses and moved to the lymphatic sinuses and cortex (Fig. 4C(a)). After US was applied to the PALN for 60 sec (Fig. 4C(b)), the echogenicity decreased (Fig. 4C(c)) in comparison to the value before exposure to US.

**Figure 5:**
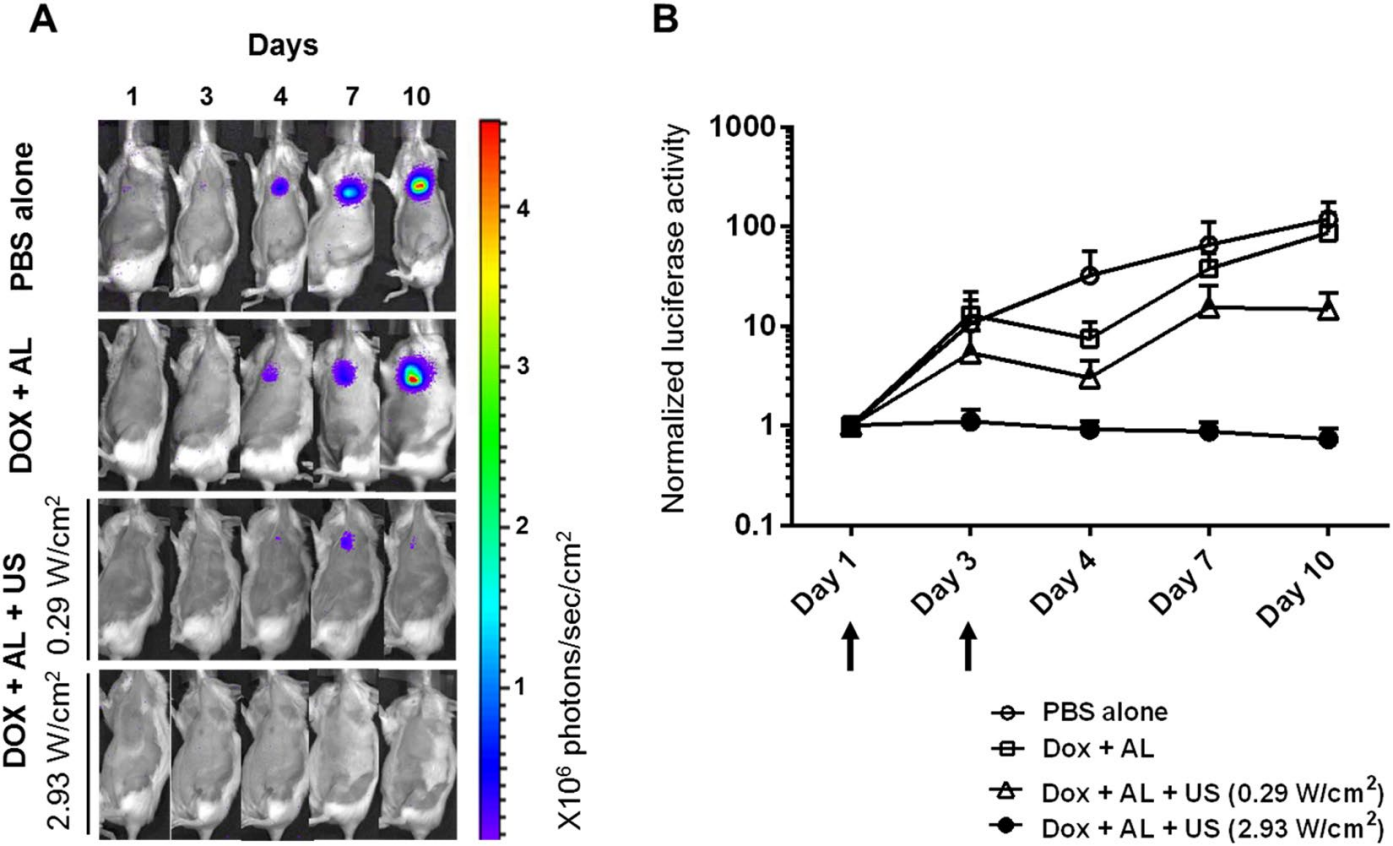
Anti-tumor effects of sonoporation in the tumor-bearing proper axillary lymph node (PALN). (**A**) *In vivo* bioluminescence imaging of mice treated with phosphate-buffered saline (PBS alone; *n* = 5), doxorubicin plus acoustic liposomes (DOX + AL; *n* = 4), DOX + AL + US (*I*_SPTA_ = 0.29 W/cm^2^; *n* = 6) and DOX + AL + US (*I*_SPTA_ = 2.93 W/cm^2^; *n* = 5). In the PBS alone and DOX + AL groups, strong bioluminescence was observed on day 10 indicating the presence of tumor cells in the PALN. In the sonoporation-treated groups, relatively low (*I*_SPTA_ = 0.29 W/cm^2^) or very little (*I*_SPTA_ = 2.93 W/cm^2^) bioluminescence was detected on day 10. (**B**) Time-dependent changes in luciferase activity in the tumor-bearing PALN. Values at different time points were normalized to those obtained on day 1. The treatment intervention was carried out on days 1 and 3 (arrows). Error bars represent the standard error of the mean. In the DOX + AL + US (*I*_SPTA_ = 2.93 W/cm^2^) group, luciferase activity remained at a low level throughout the experiment, while the other groups exhibited progressive increases in luciferase activity.

Temporal changes in luciferase activity in the tumor-bearing PALN were quantified to verify the anti-tumor effects of our technique. Figure 5A shows representative bioluminescence images at various time points. In the groups treated with phosphate-buffered saline (PBS) alone or DOX combined with ALs (DOX + AL), the bioluminescence in the PALN increased progressively and reached a maximum level on day 10, which was the endpoint of the experiment. A smaller increase in bioluminescence was detected in the DOX + AL + US (0.29 W/cm^2^) group, which was administered DOX plus ALs and then exposed to low-power US (0.29 W/cm^2^). Notably, hardly any increase in bioluminescence occurred in the DOX + AL + US (2.93 W/cm^2^) group, which was exposed to high-power US (2.93 W/cm^2^). Figure 5B shows the temporal changes in luciferase activity normalized to the value on day 1. The luciferase activity in the DOX + AL + US (2.93 W/cm^2^) group was maintained at a low level throughout the experiment, although statistical significance in comparison to the other groups was not detected at any time points.

### Histological evaluation of the anti-tumor effect of lymphatic drug administration combined with sonoporation

**Figure 6:**
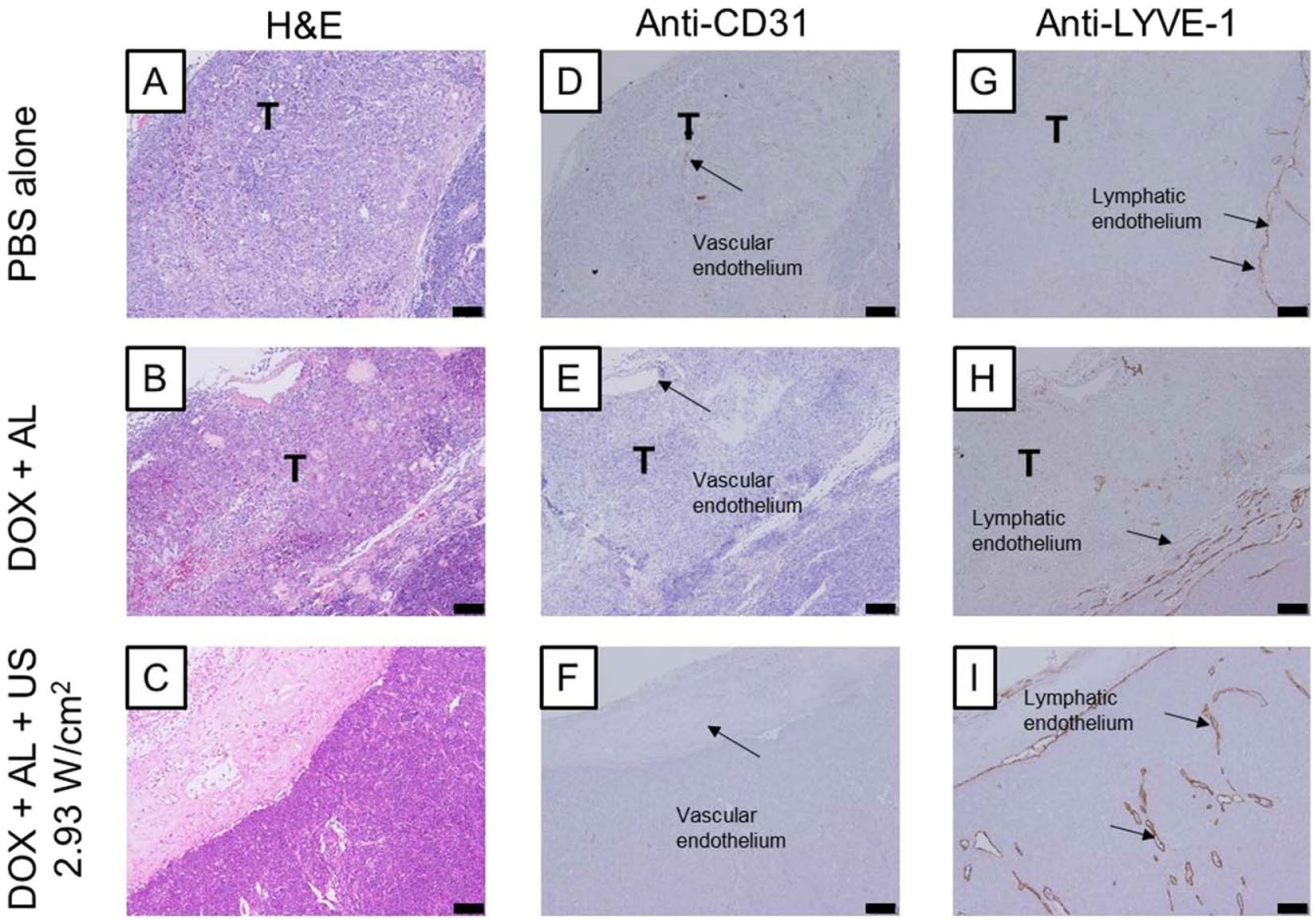
Histological analysis of paraffin sections of the proper axillary lymph node (PALN) on day 10. (**A**–**C**) Hematoxylin/eosin (H&E) staining. (**D**–**F**) Anti-CD31 staining. (**G**–**I**) Anti-LYVE-1 staining. (**A**, **D**, **G**) Phosphate-buffered saline (PBS) alone group. (**B**, **E**, **H**) Doxorubicin plus acoustic liposomes (DOX + AL) group. (**C**, **F**, **I**) DOX + AL + US (*I*_SPTA_ = 2.93 W/cm^2^) group. There were no differences between the three groups in the internal structure of the PALN, indicating that the mechanical effects of sonoporation did not induce significant tissue damage. (**A**) Tumor proliferation was observed at the sites corresponding to the marginal sinuses, but extranodal infiltration did not occur. (**D**) CD31-positive vascular endothelia were observed in the tumor tissues growing at the sites corresponding to the marginal sinuses, but noticeable progression of newly formed vessels was not confirmed. (**G**) LYVE-1-positive lymphatic endothelia were not observed in the tumor tissues growing at the sites corresponding to the marginal sinuses. LYVE-1-positive littoral cells covering the inner surface of the marginal sinuses had virtually disappeared from the regions containing growing tumor. (**B**) Tumor proliferation was observed at the sites corresponding to the marginal sinuses, but extranodal infiltration was not detected. Necrotic foci and tumor cell loss were sporadically observed within the tumors. (**E**) CD31-positive vascular endothelia were not observed in the tumor tissues growing at the sites corresponding to the marginal sinuses. (**H**) A clear increase in LYVE-1-positive lymphatic endothelia could not be confirmed in the tumor tissues growing at the sites corresponding to the marginal sinuses. LYVE-1-positive littoral cells covering the inner surface of the marginal sinuses had virtually disappeared from the regions containing growing tumor. (**C**) Necrosis of tumor tissue was observed at the sites corresponding to the marginal sinuses. Residual tumor cells could not be confirmed, while the structure of the capsule of the PALN was preserved. (**F**) CD31-positive vascular endothelia in the necrotic region corresponding to the marginal sinuses were not observed. (**I**) LYVE-1-positive lymphatic endothelia in the necrotic region corresponding to the marginal sinuses were not observed, but the structure of the capsule of the PALN was preserved, and LYVE-1-positive endothelia, thought to be littoral cells covering the inner surface of the marginal sinuses, were detected. The symbol “T” represents a tumor region in the marginal sinus. Scale bar: 100 μm.

Finally, histopathological analyses of the tumor-bearing PALN at day 10 were carried out to evaluate the anti-tumor effects and potential tissue damage induced by our treatment method. Figure 6 shows representative microscopic images of the tumor-bearing PALN stained with hematoxylin/eosin (H&E; Fig. 6A–C) or immunostained for CD31 (Fig. 6D–F) or LYVE-1 (Fig. 6G–I). In the PBS group, the proliferation of FM3A-Luc cells was observed in the marginal sinus, which contained CD31-positive vascular endothelia, but neovascularization or extranodal infiltration of tumor cells could not be confirmed (Fig. 6A, B). In the DOX + AL group, the proliferation of FM3A-Luc cells was also observed in the marginal sinuses, which had CD31-positive vascular endothelia and neovascularity, but extranodal infiltration could not be confirmed.

Additionally, sporadic necrotic foci or regions of cell loss within the tumor lesion were observed in the marginal sinus (Fig. 6B, E). In the DOX + AL + US group, anuclear necrotic foci were observed, but residual tumor cells were not detected in the marginal sinus (Fig. 6C). Similarly, CD31-positive vascular endothelia were not found in the necrotic foci (Fig. 6F). In the PBS and DOX + AL groups, a clear increase in LYVE-1-positive lymphatic endothelia could not be confirmed within the tumor tissues in the marginal sinus, and LYVE-1-positive littoral cells covering the inner surface of the marginal sinus had virtually disappeared (Fig. 6G, H). In the DOX + AL + US group, LYVE-1-positive lymphatic endothelia were not observed in the necrotic region in the marginal sinuses, but the structure of the capsule of the PALN was preserved, and LYVE-1-positive endothelia (believed to be littoral cells covering the inner surface of the marginal sinus) were present (Fig. 6I).

## Discussion

Here we report that lymphatic drug administration combined with sonoporation resulted in enhanced anti-tumor effects against breast cancer cells proliferating in the marginal sinus of the PALN. Selective lymphatic administration was achieved by the injection of a solution into one of the swollen LNs of the MXH10/Mo/lpr mouse, which is a unique animal model system (Fig. 1A, B). Solutions or drugs injected into the SiLN flowed through the LVs and entered the tumor-bearing PALN (Fig. 1C, D). We confirmed that the ALs flowed via the LVs into the marginal sinus of the PALN and reached the cortex (Fig. 2). Even 5 days after tumor cell inoculation, solutions injected into the SiLN could access the tumor lesions in the PALN via the LVs (Fig. 3). If US was applied to the PALN after the injection of a drug with ALs, the echogenicity in the PALN was reduced due to collapse of the ALs (Fig. 4). This technique led to suppression of tumor proliferation throughout the experimental period without notable damage to normal tissues (Figs 5 and 6).

One of the intriguing findings of the present study is that the littoral cells in the marginal sinus of the tumor-bearing PALN were preserved following lymphatic drug administration and application of US (Fig. 6I), whereas these cells were diminished in number in the negative control group (Fig. 6G) or after the administration of drugs without US (Fig. 6H). This finding suggests that in the latter two groups, residual tumor cells were able to proliferate in the marginal sinus and erode the littoral cells. When tumor cells spread to the sentinel LNs through the LVs, the metastatic cells first reach the marginal sinuses^18^. However, the mechanisms by which metastatic cells subsequently spread to distant organs remain to be elucidated. Some research groups have reported that metastatic cells from the primary tumor enter the blood circulation via the blood vessels of the sentinel LN^19,20^. One group reported that metastatic cells invaded the inner region of the sentinel LN to reach the high endothelial venules, resulting in pulmonary metastasis^20^. Although the authors stated that metastatic tumor cells in the sentinel LN did not leave via efferent LVs in their animal model, another research group argued that overexpression of vascular endothelial growth factor (VEGF)-C in the cell line allowed the tumor cells to metastasize to the downstream LN^21^, while overexpression of VEGF-A was reported to contribute to distant metastasis via the blood vessels of the sentinel LN rather than through lymphatic routes^22^. The FM3A cells utilized in the present study express VEGF-A but not VEGF-C^23^, hence these tumor cells may spread to distant organs via the blood vessels after their arrival in LNs such as the PALN. Indeed, our study detected CD31-positive blood vessels in regions of tumor proliferation, and this may have enabled residual tumor cells to enter the blood circulation (Fig. 6D, E).

The invasion of tumor cells into the central region of a LN necessitates the delivery of drugs into this deeper region of the node. During conventional hematogenous administration, US contrast agents like microbubbles can diffuse throughout the body and be diluted by the blood or expelled by the lungs. Lymphatic administration by intranodal injection retains drugs in the lymphatic network for a relatively long duration because the lymphatic network lacks an independent driving pump, and the sheer force of a LV is relatively strong^6^. In the present study, lymphatic administration of ALs resulted in a high-intensity signal in the PALN (Fig. 2). Notably, the ALs delivered from the afferent LVs were located around the marginal sinus in the PALN, which is the region invaded by metastatic cells^24^. Echogenicity was detected around the cortex of the PALN 90 sec after the injection of ALs and was maintained stably (Fig. 2). In our previous study^6^, the signal intensity of ALs and/or gas vesicles from ALs reached a maximum value at 2 min and then gradually decreased, although the signal remained at a high value for more than 6 min after reaching its maximum. When fluorescent molecules (indocyanine green; ICG) were injected into the SiLN, fluorescence in the PALN was detected within a few minutes and could be observed for 6 hours after the injection^25^. These observations reveal that a high concentration of ALs can reach the cortex of the PALN and remain for longer than that achieved by systemic administration^6^. In our previous study, tumor cells growing in the PALN were able to block the flow from the upstream SiLN^25^. In the present study, India ink from the upstream SiLN was demonstrated to reach the lesion in the tumor-bearing PALN, indicating that the structure of the LVs in the PALN was preserved even 5 days after tumor inoculation (Fig. 3). In relation to this, our colleagues have previously investigated the biodistribution of fluorescent ICG after its administration via blood vessels or LVs^26^. Both routes of administration resulted in ICG accumulation in the liver, but hepatic accumulation of ICG was greater after systemic administration than after lymphatic administration. By contrast, the accumulation of ICG in the PALN was greater for lymphatic administration than for systemic administration. This implies that a high concentration of drug can reach the target LNs after lymphatic administration, which in turn would be predicted to result in a good clinical response to the drug. Furthermore, a high concentration of ALs would also be expected to reach the target LNs after lymphatic administration, enabling the ALs to act as cavitation nuclei in the US field.

The interaction of lymphatically administered ALs in the PALN with US could affect cell membrane permeability. Tomita *et al*. characterized the behavior of sonazoid microbubbles, including C_4_F_10_ gas and vapored C_4_F_10_ gas, in the presence of 1-MHz focused US^27^. In the initial phase of irradiation, ALs or C_3_F_8_ gas could collapse and cause inertial cavitation, inducing shear stress due to microstreaming or impulsive pressures due to liquid jets. These phenomena can loosen cell-cell adhesion in lymphatic endothelia^11^. Further exposure of C_3_F_8_ gas to US was able to induce the development of inertial cavitation bubbles, leading to drug extravasation into the tumor region in the PALN^5,6^. An increasing number of pores are generated in the cell membrane during the process of cavitation bubble oscillation and collapse, which induces a transient enhancement of cell membrane permeability^28,29^. In short, these mechanical pressures might lead to drug extravasation out of the LVs and efficient drug delivery to the tumor cells, resulting in strong anti-cancer effects. This means that highly invasive tumor cells proliferating in the marginal sinuses could be effectively targeted by lymphatic drug administration with sonoporation.

In Fig. 2C, the echogenicity of ALs in the marginal sinuses of the PALN was first detected around 1 min after injection. When the echogenicity of the ALs reached a maximum value at 2 min, the ALs flowed from the marginal sinuses into deeper structures, namely the lymphatic sinuses. 3-D HF-US imaging allowed the volume of the PALN and the LV area in the PALN to be estimated (Table 1). Based on these values, the LV volume in the PALN was about 58 mm^3^ (μL). Unfortunately, we did not acquire data for SiLN volume. However, provided that the volume of the SiLN is similar to that of the PALN, the PALN would be predicted to fill with ALs and DOX after the injection of around 100 μL of solution (2 min after injection). In our previous study^5^, we investigated the DOX distribution in the tumor-bearing PALN and revealed that co-administration of DOX + AL through LVs combined with US was superior in DOX potency and tissue selectivity to the condition of a single administration of DOX. Little DOX fluorescence was detected in the PALN after a single administration of DOX half an hour after administration, indicating that DOX in the PALN went through efferent LVs or blood vessels by degrees. While there might be differences in sizes and some physicochemical properties between the ALs and DOX, the concentration of DOX and the ALs (Fig. 2C) in the PALN could be maximal in the PALN immediately after finishing the administration. In the present study, as US was applied to the PALN immediately after finishing the administration, abundant cavitation bubbles would be generated in the high concentration field of DOX, resulting in high anti-tumor effects. The cytotoxicity of lymphatic drug administration with sonoporation was evaluated based on alterations in mouse body weight, and no significant body weight changes were detected during the experiments (Supplementary Fig. 1). We have already confirmed that lymphatic administration with sonoporation has minimal systemic toxicity^5^. Our previous studies did not detect any acute toxic effects of lymphatically-administered cisplatin on hepatic or renal function^26,30^. Furthermore, histopathological evaluation did not show evidence of structural changes or additional inflammation in the DOX + AL + US group as compared with the PBS alone or DOX + AL groups (Fig. 6A, B respectively).

Occasionally, surgical treatments such as intranodal injection can lead to lymphorrhea, which is a lymphatic complication related to postoperative lymphatic exudation^31^. Precise and efficient intranodal injection is critical to minimize the leakage of lymph and prevent postoperative adverse events. Although the present study did not detect echogenicity in the TEV after the intranodal injection of ALs (Fig. 1B–D), it remains likely that a small proportion of the drug had leaked outside the SiLN. High lymphatic pressure due to intranodal injection may contribute to lymphatic leakage^8^. In the present study, lymphorrhea was not detected during the 10-day experimental period, although it may take longer for this adverse effect to develop. Therefore, adapting our proposed method for use in clinical practice will necessitate further investigation of the relationship between the injection rate/volume and the onset of lymphatic complications such as lymphorrhea.

In conclusion, lymphatic administration permits drugs to access tumor lesions in a tumor-bearing LN and remain in the LN for an extended period. Furthermore, the lymphatic administration of ALs and subsequent exposure to US might generate mechanical pressures that facilitate drug delivery to highly invasive breast cancer cells in the marginal sinus of the LN. We believe that the technique described in this study has the potential to be developed into a new treatment for LNs invaded by metastatic tumor cells.

## Methods

All experiments were carried out in accordance with approved guidelines and were approved by the Institutional Animal Care and Use Committee of Tohoku University. For the *in vivo* experiments, mice were anesthetized with 2% isoflurane (Abbott Japan Co., Ltd., Tokyo, Japan) using an inhalation gas anesthesia system for small laboratory animals.

### Cell preparation

FM3A (murine mammary carcinoma) cells were established from the C3H/He mouse and obtained from the Cell Resource Center for Biomedical Research, Institute of Development, Aging and Cancer, Tohoku University. FM3A-Luc cells, which stably expressed the firefly luciferase gene, were established by electroporation of FM3A cells with pGL4.51 (Invitrogen, Carlsbad, CA, USA) using a Gene Pulser Xcell (Biorad, Hercules, CA, USA). FM3A-Luc cells were cultured in RPMI 1640 medium (Biological Industries, Kibbutz Beit Haemek, Israel), supplemented with 10% fetal bovine serum (FBS; Thermo Fisher Scientific, Waltham, MA, USA), 1% penicillin/streptomycin (Sigma-Aldrich, St. Louis, MO, USA) and 0.5 mg/mL geneticin (G418 sulfate, Sigma-Aldrich). Cells were incubated at 37 °C in an atmosphere of 5% carbon dioxide and 95% air. Before experiments commenced, the absence of *Mycoplasma* contamination in the cell cultures was ensured using a MycoAlert Mycoplasma Detection Kit (Lonza Rockland, Inc., Rockland, ME, US), according to the manufacturer’s protocol.

### Animal model

MXH10/Mo/lpr mice are a substrain of the recombinant inbred mouse strain, MXH/*lpr*/*lpr* (MXH/lpr). MXH10/Mo/lpr mice were produced using two different parental inbred strains as progenitors, MRL/MpJ-*lpr*/*lpr* (MRL/lpr; the major histocompatibility complex is H-2^k^) and C3H/HeJ-*lpr*/*lpr* (C3H/lpr; H-2^k^), followed by an F1 intercross and more than 20 generations of strict brother-sister mating. MXH10/Mo/lpr mice are unique, and the size of most peripheral LNs is up to 10 mm at 2.5 to 3 months of age (Fig. 1A). It is noteworthy that MXH10/Mo/lpr mice develop only mild autoimmune diseases^32^. MXH10/Mo/lpr mice do not express the fas gene involved in apoptosis, since the lpr gene is a fas-deletion mutant gene. Thus, the immune system in MXH10/Mo/lpr mice is functional except for the signaling pathway related to fas^33^, which is not associated with tumor growth in LNs. Thus, we can avoid using unrepresentative models of LN metastasis based on nude or SCID mice that exhibit immune system failure. MXH10/Mo/lpr mice have a longer lifespan than MRL/lpr mice in part because they do not develop glomerulonephritis, vasculitis, arthritis and sialadenitis, and this is an additional advantage for LN metastasis experiments^32^. Furthermore, as the sizes of the LNs in MXH10/Mo/lpr mice are similar to those in humans, it is more likely that the mechanisms underlying tumor spread and treatment responses reflect those in patients in the clinical setting. Given the above points, we consider the MXH10/Mo/lpr mouse to be a good model of LN metastasis.

### Nano/microbubbles

ALs were used as nano/microbubbles; these were composed of 1,2-distearoyl-*sn*-glycero-3-phosphatidylcholine (DSPC; NOF Co., Tokyo, Japan) and 1,2-distearoyl-*sn*-glycero-3-phosphoethanolamine-methoxy-polyethyleneglycol (DSPE-PEG[2000-OMe]; NOF Co.) (94:6 mol/mol), containing C_3_F_8_ gas, as described previously^34,35^. The particle size of the ALs was 199 ± 84.4 nm, and the zeta potential was −2.10 ± 0.90 mV. Approximately 20% of the ALs contained both liquid and gas, whereas approximately 80% contained only liquid.

### Measurement of US parameters

Signals of 970 kHz were generated by a multifunction synthesizer (WF1946A; NF Co., Yokohama, Japan) and amplified with a high-speed bipolar amplifier (HSA4101; NF Co.). The peak negative pressure *P*_–_ and the intensity *I*_A_ of the US were measured by a PVDF needle hydrophone (PVDF-HNP-1100; Specialty Engineering Associates, Soquel, CA, US) at a 1-mm stand-off distance from the transducer surface using a three-dimensional (3-D) stage control system (Mark-204-MS; Sigma Koki, Tokyo, Japan). Calibration of the US pressures was performed as previously described^5^. The spatial peak-temporal average intensity could be determined by the equation:

1 $${I}_{SPTA}=\frac{{(2{P}_{A})}^{2}}{8\rho c}\times (duty\,ratio)$$

where ρ is the density of water (1,000 kg/m^3^ at 20 °C), and c (1,479 m/s at 20 °C) is the sound velocity of water. Ultrasound parameters used in the experiments are summarized in Table 2. The experiments used *P* = 0.21 (MPa) and 0.67 (MPa).

**Table 2:** Ultrasound parameters.

*P*_–_ (MPa)	*I*_A_ (W/cm^2^)	Duty ratio (%)	*I*_SPTA_ (W/cm^2^)	Exposure time (sec)
0.21	3	20	0.29	60
0.67	30	20	2.93	60

*P*_–_: peak negative pressure, *I*_A_: ultrasound intensity, *I*_SPTA_: spatial peak-temporal average intensity.

### Visualization of ALs flowing into LVs and the PALN by contrast-enhanced HF-US imaging

With the mouse in the lateral position, ALs were injected into the SiLN to deliver them to the PALN via the LVs. A 27 G butterfly needle (Terumo, Tokyo, Japan), attached to a 1-mL syringe (Terumo) containing ALs, was inserted into the SiLN under the guidance of contrast-enhanced HF-US. Figure 1B shows the SiLN (a) before and (b) after the injection of ALs.

Two female mice (15 or 17 weeks old; lateral position) were used to observe the dynamics of the flow of ALs into the LVs. After the butterfly needle was inserted into the SiLN, the contrast-enhanced HF-US imaging system, which was equipped with a mechanical single-element transducer (RMV-708; central frequency, 55 MHz; axial resolution, 30 μm; lateral resolution, 70 μm; focal length, 4.5 mm; depth of field, 1.4 mm; VisualSonics, Toronto, ON, Canada), was fixed to an appropriate lateral site in close proximity to the TEV. B-mode images were obtained immediately after the injection of ALs at a rate of 50 μL/min, and echogenicity was analyzed as previously described^6^.

### Construction of a 3-D image of the PALN and quantification of its LV density

Five female mice (15–21 weeks old) were used in experiments quantifying the LV density of the PALN by contrast-enhanced HF-US. First, B-mode images of the whole length of the PALN were scanned as a reference. Next, 150 μL of ALs was injected into the SiLN at a rate of 50 μL/min, and the entire length of the PALN was scanned as previously described^36^. Increased echogenicity derived from the ALs was regarded as LVs in the PALN and marked in green. The LV density was defined as the ratio of the volume of LVs to the volume of the PALN.

### Visualization of the lymphatic channel in the PALN inoculated with tumor cells

Mice were used for visualization of the lymphatic channel on day 3 (*n* = 1) and day 5 (*n* = 2) after inoculation of tumor cells into the SiLN. India ink (50 μL) was injected into the PALN with a butterfly needle at a rate of 50 μL/min, and then immediately the PALN was surgically removed and frozen in liquid nitrogen. The frozen samples were sectioned (10 μm thickness) with a cryostat (Thermo Fisher Scientific, Barrington, IL, US) and stained with H&E.

### Development of the tumor-bearing LN mouse model

A tumor-bearing PALN was induced by the injection of 4.0 × 10^4^ FM3A-Luc cells, suspended in a mixture of 13 μL PBS and 26 μL of 400 mg/mL Matrigel (Collaborative Biomedical Products), into the SiLN of the mouse. Cell inoculation was guided by the HF-US imaging system with a 25-MHz transducer (RMV-710B; axial resolution, 70 μm; focal length, 15 mm; VisualSonics).

### *In vivo* treatment of the tumor-bearing PALN using lymphatic drug delivery and sonoporation

Doxorubicin (Wako) was used as an anti-tumor agent. Mice (16–18 weeks old) were divided into 4 groups: PBS alone (control, *n* = 5), DOX + AL (*n* = 4), DOX + AL + US(0.29 W/cm^2^) (*n* = 6) and DOX + AL + US(2.93 W/cm^2^) (*n* = 5). The concentration of doxorubicin was 5 mg/kg and that of the ALs was 1 mg/mL. The solutions were delivered into the SiLN at a rate of 50 μL/min. For the groups exposed to US, the tumor-bearing PALN was subsequently exposed to US as previously described^5,6^. Briefly, US was applied to the tumor-bearing PALN immediately after finishing the administration when the concentration of DOX and ALs could reach a maximum value. Figure 4A shows a schematic diagram of the US exposure treatment. The position of the tumor-bearing PALN, flow of ALs in the PALN (Fig. 4B(a)) and US exposure (Fig. 4B(b)) were confirmed using a contrast-enhanced HF-US imaging system with a mechanical single-element transducer (RMV-710B; central frequency, 25 MHz; axial resolution, 70 μm; lateral resolution, 140 μm; focal length, 15 mm; depth of field, 2.7 mm). To confirm the destruction of the ALs by US, the grayscale intensity in the area representing the PALN (dotted line in Fig. 4B) was calculated by VEVO software (VisualSonics). Treatment was carried out on days 1 and 3 after tumor inoculation (the day of inoculation being defined as day 0); the irradiation conditions are listed in Table 2.

### Monitoring of tumors in the PALN by measurement of luciferase activity

To evaluate the therapeutic effect in each group, *in vivo* bioluminescence imaging was performed using an IVIS Lumina system (Xenogen Co., Alameda, CA, US). Luciferase activity was quantified on days 1, 3, 4, 7 and 10. Each mouse was injected intraperitoneally with 150 μg/g body weight of luciferin (*Promega*, Sunnyvale, CA, US), and 10 min after administration the light emitted by the luciferase was measured for 30 sec. The luciferase activity was normalized to that measured on day 1.

### Histological analysis

After treatment on day 10, the PALNs were extracted and fixed overnight in 10% formaldehyde in PBS at 4 °C for 4 days, dehydrated and then embedded in paraffin. The embedded specimens were cut into 4-μm serial sections and either stained with H&E or immunostained for detection of LYVE-1-positive and CD31-positive cells using a Discovery XT automated staining processor (Ventana Medical Systems, Inc., Tucson, AZ, USA). Immunostaining of lymphatic endothelial cells was performed using a polyclonal rabbit anti-mouse LYVE-1 antibody (4 μg/ml; 103-PA50AG, Cosmo Bio Co. Ltd., Tokyo, Japan) for 2 h at room temperature, an anti-rabbit IgG Histofine MAX-PO (R) kit (Nichirei Biosciences Inc., Tokyo, Japan) for 16 min at room temperature and diaminobenzidine (DAB). Immunostaining of vascular endothelial cells was carried out using a pre-diluted polyclonal rabbit anti-CD31 antibody (1/100 dilution; sc-1506-R, Santa Cruz Biotechnology, Inc., CA, US) for 2 h at room temperature in combination with an anti-rabbit IgG Histofine MAX-PO (R) kit for 16 min at room temperature.

### Statistical analysis

All measurements are expressed as the mean ± SD or SE. Any overall difference between groups was determined by one-way ANOVA. Statistical comparisons were performed using Prism 6.0 software (GraphPad Prism). Differences were considered to be significant at *P* < 0.05.

## Supplementary information

supplementary info
