# Supplementary material for: Use of a Lymphatic Drug Delivery System and Sonoporation to Target Malignant Metastatic Breast Cancer Cells Proliferating in the Marginal Sinuses
Source: Sci Rep. 2019 Sep 13;9:13242. doi: 10.1038/s41598-019-49386-5 (PMC6744402; doi:10.1038/s41598-019-49386-5)
Supplement: Supplementary file 1 — supplementary info [file 41598_2019_49386_MOESM1_ESM.pdf]

Supplementary Info File

Use of a Lymphatic Drug Delivery System and Sonoporation to Target Malignant Metastatic Breast Cancer Cells Proliferating in the Marginal Sinuses

Shigeki Kato[1, 2, 3], Yuko Shirai[1], Maya Sakamoto[4],

Shiro Mori[1, 2, 5], Tetsuya Kodama[1]

[1] Laboratory of Biomedical Engineering for Cancer, Graduate School of Biomedical Engineering, Tohoku University, 4-1 Seiryo, Aoba, Sendai, Miyagi 980-8575, Japan

[2] Biomedical Engineering Cancer Research Center, Graduate School of Biomedical Engineering, Tohoku University, 4-1 Seiryo, Aoba, Sendai, Miyagi 980-8575, Japan

[3] Department of Immunology, Kindai University Faculty of Medicine, Osaka-Sayama, Osaka 589-8511, Japan

[4] Department of Oral Diagnosis, Tohoku University Hospital, 1-1 Seiryo, Aoba, Sendai, Miyagi 980-8575, Japan

[5] Department of Oral Medicine and Surgery, Tohoku University Hospital, 1-1 Seiryo, Aoba, Sendai, Miyagi 980-8575, Japan

Corresponding author: Tetsuya Kodama, PhD (Eng), PhD (Med), Laboratory of Biomedical Engineering for Cancer, Graduate School of Biomedical Engineering, Tohoku University, 4-1 Seiryo, Aoba, Sendai, Miyagi 980-8575, Japan. Tel & Fax: +81-22-717-7583; E-mail: kodama@tohoku.ac.jp

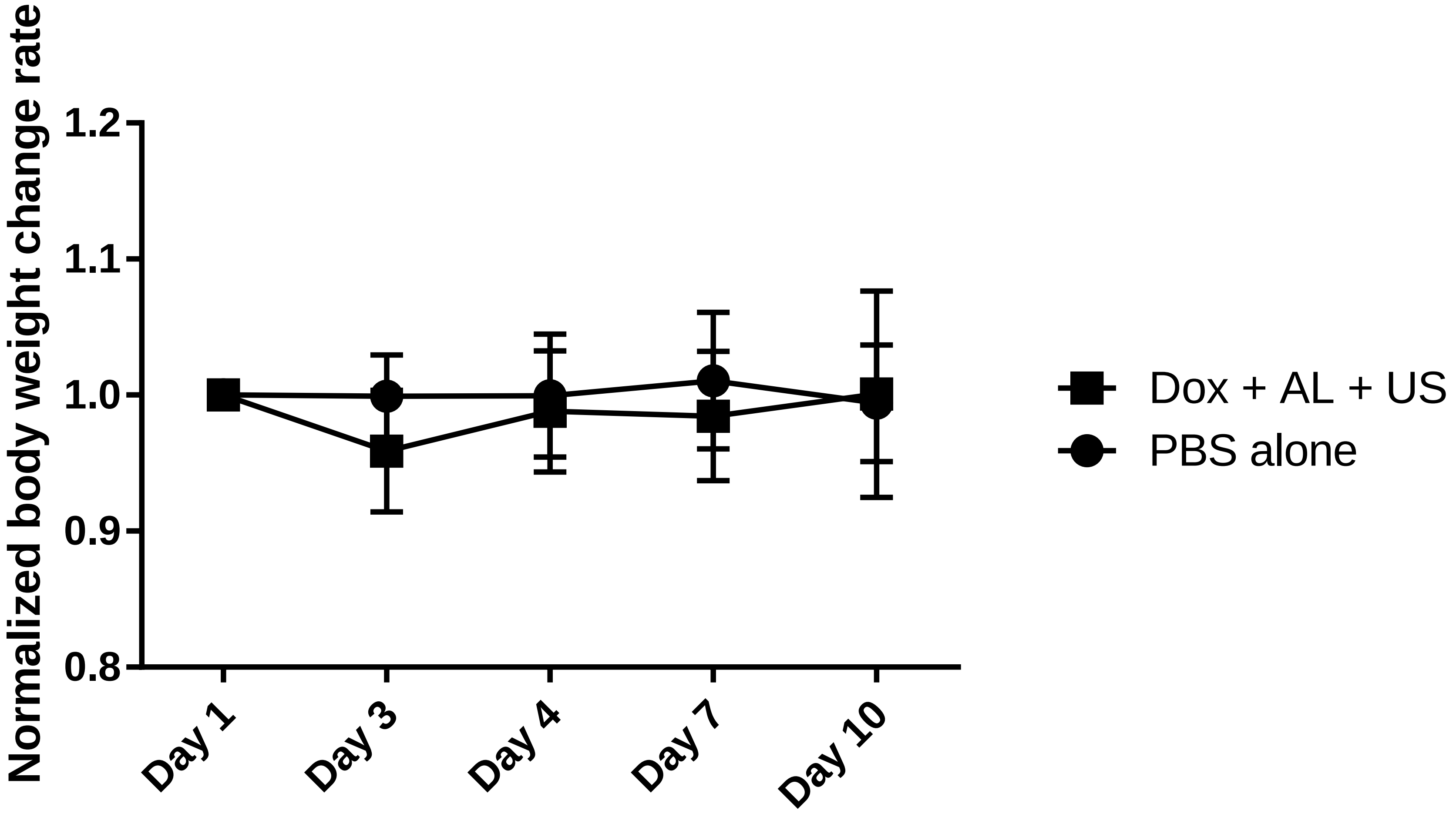

Normalized body weight change rate
1.2
1.1
1.0
0.9
0.8
Day 1
Day 3
Day 4
Day 7
Day 10
Dox + AL + US
PBS alone

Supplementary Figure 1

Temporal changes in mouse body weight normalized to the value obtained on day 0 before cell inoculation. PBS alone (n = 5), Dox+AL+US (n = 5). Error bars represent the SD. Statistical comparisons were made using one-way ANOVA and a Tukey-Kramer post-hoc test. No significant changes in body weight were detected.
